# A Rare Case of Mediastinal Seminoma with Granulomatous Features: Diagnostic and Therapeutic Considerations

**DOI:** 10.70352/scrj.cr.25-0091

**Published:** 2025-07-01

**Authors:** Ryusei Yoshino, Nanami Ujiie, Shunsuke Yasuda, Yuki Kamikokura, Masahiro Kitada

**Affiliations:** 1 Department of Thoracic Surgery and Breast Surgery, Asahikawa Medical University Hospital, Asahikawa, Hokkaido, Japan; 2 Department of Diagnostic Pathology, Asahikawa Medical University Hospital, Asahikawa, Hokkaido, Japan

**Keywords:** seminoma, anterior mediastinum, granulomatous, immunohistochemistry

## Abstract

**INTRODUCTION:**

Although seminomas typically arise in the testes, primary mediastinal seminomas are classified as extragonadal germ cell tumors. Diagnosis is often challenging and requires not only blood tests and imaging but also a tumor biopsy. However, diagnosis may be particularly difficult when the tumor shows nonspecific pathological features or is accompanied by granulomatous changes.

**CASE PRESENTATION:**

The patient was a 25-year-old man who had been experiencing labored breathing when leaning forward for the past month. Physical examination revealed distended jugular veins and neck edema. Chest computed tomography revealed an irregular mass measuring 80 mm in the anterior mediastinum, suggesting invasion of the superior vena cava. Additionally, fluorodeoxyglucose-positron emission tomography showed high accumulation in the same area, with a maximum standardized uptake value of 11.3. A tumor biopsy was performed under thoracoscopic guidance for definitive diagnosis. Histopathological examination of the resected specimen revealed a seminoma with granulomatous changes. Based on these findings, a diagnosis of anterior mediastinal seminoma with superior vena cava syndrome was made. It was classified as having a good prognosis, and the patient received three courses of induction chemotherapy with etoposide, cisplatin, and ifosfamide. Complete remission was achieved. Since then, the patient has been monitored every 3 months, with no recurrence or metastasis observed for approximately 2 years.

**CONCLUSIONS:**

Immunohistochemical analysis plays a crucial role in the accurate diagnosis of mediastinal seminomas, especially in cases with unusual histological features such as granulomatous changes. Recognizing the immunoprofile of seminomas and differentiating them from thymomas and lymphomas is essential for avoiding diagnostic pitfalls.

## Abbreviation

CT
: computed tomography

## INTRODUCTION

Although seminomas generally occur as testicular tumors, they can also originate in the mediastinum, where they are classified as germ cell tumors.^1)^ Primary anterior mediastinal seminoma is a relatively rare disease that primarily affects males in their teens and thirties.^2)^ Diagnosis is often challenging and requires a combination of tumor biopsy, blood tests, and imaging. However, it can be particularly difficult in cases where the tumor exhibits nonspecific pathological features or is accompanied by granulomatous changes.^3)^

## CASE PRESENTATION

The patient was a 25-year-old male, 179 cm tall, weighing 62.2 kg, with a body mass index of 19.4 kg/m^2^. The patient had no history of smoking. His medical history showed that he had undergone surgery for a varicocele 1 year prior. Respiratory function test results were as follows: vital capacity, 4530 mL; 1-s volume, 4020 mL; with no obstructive or restrictive ventilatory impairment. No abnormal electrocardiogram findings were observed.

The patient had noticed labored breathing when leaning forward for the past month. A previous physician had detected a tumor in the anterior mediastinum and referred the patient to our department for further examination. Physical examination revealed distended jugular veins and neck edema. There was no mass in the anterior chest and no enlargement of the cervical and subclavian lymph nodes. Additionally, no facial edema or cyanosis of the extremities was observed.

**Fig. 1:**
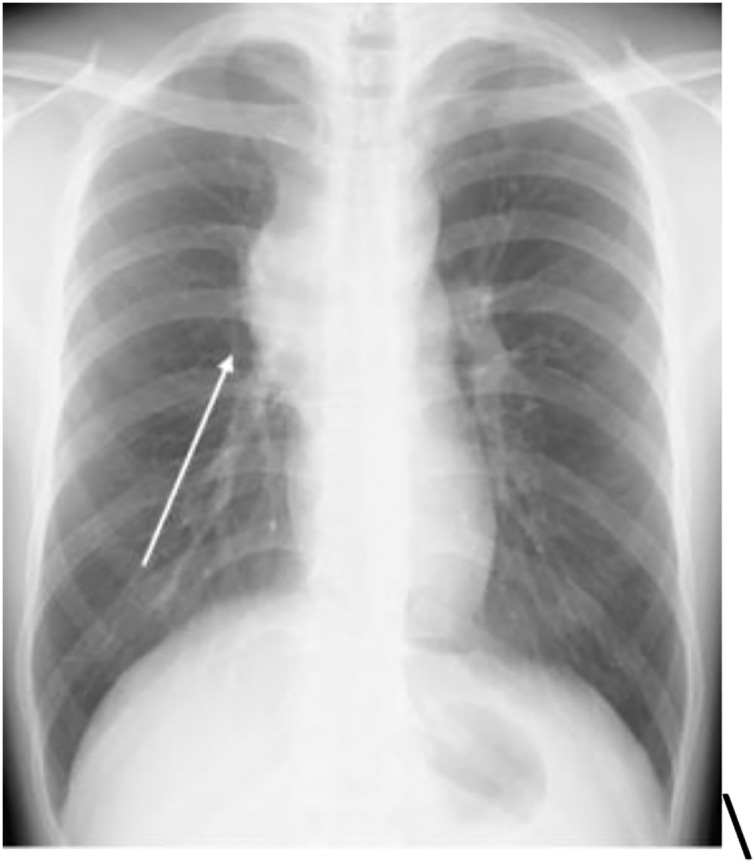
Chest radiograph (frontal view) showing a suspected tumor in the anterior mediastinum (arrow).

**Fig. 2:**
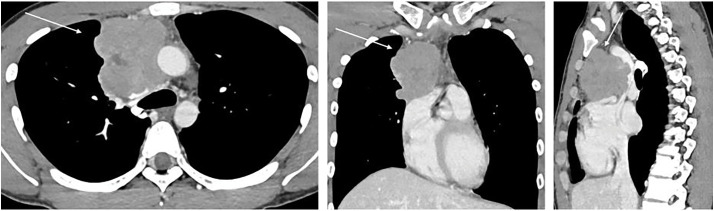
Chest computed tomography findings. An irregular mass measuring 80 mm was detected in the anterior mediastinum, with findings suggestive of invasion of the superior vena cava (arrow).

**Fig. 3:**
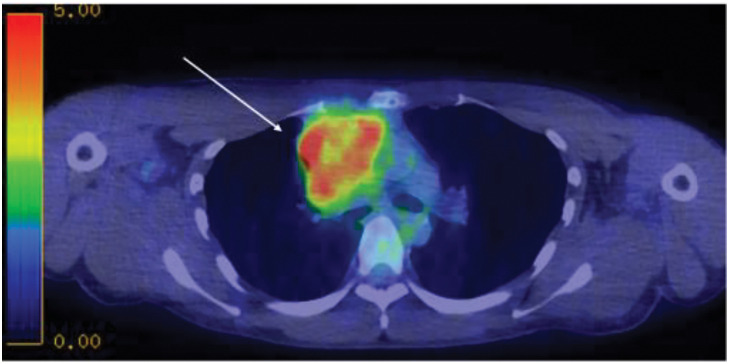
^18^F-fluorodeoxyglucose-positron emission tomography findings. A high level of accumulation (Max SUV 11.3) was observed in the indicated anterior mediastinal tumor (arrow). SUV, standardized uptake value

Blood count and biochemical test results were within normal limits. The serum human chorionic gonadotropin level was 1.83 mIU/mL (<2.0 mIU/mL), and the α-fetoprotein level was 2 ng/mL (<10 ng/mL), both within normal limits. Lactate dehydrogenase and soluble interleukin-2 receptor levels were also normal. Tumor markers related to lung cancer showed no abnormalities. Chest radiography suggested the presence of a tumor in the anterior mediastinum (**Fig. 1**). Computed tomography (CT) revealed an irregular mass measuring 80 mm in the anterior mediastinum with findings suggestive of superior vena cava invasion (**Fig. 2**). No evidence of enlarged hilar lymph nodes was present. Fluorodeoxyglucose-positron emission tomography revealed high accumulation in the same area, with a maximum standardized uptake value of 11.3 (**Fig. 3**). Based on these findings, we suspected malignant lesions including thymomas, thymic carcinomas, and malignant lymphomas. A physical examination revealed superior vena cava syndrome. Based on the results of the above tests, a biopsy of the anterior mediastinal tumor was performed to establish a definitive diagnosis.

**Fig. 4:**
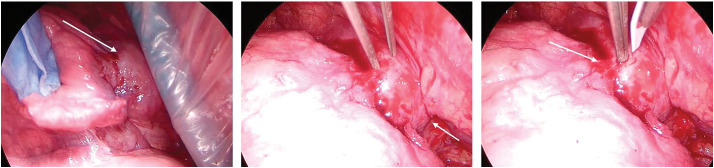
Intraoperative findings. The tumor was located in the anterior mediastinum, adhered to the upper right lung, and located above the superior vena cava (arrow). The lesion was partially separated using a sharp-pointed scalpel.

Surgery was performed with the patient in the left lateral position under general anesthesia using a thoracoscope. Observation of the thoracic cavity revealed that the anterior mediastinal tumor was adherent to the right upper lobe of the lung and located above the superior vena cava (**Fig. 4**). No pleural dissemination was observed. A portion of the tumor was excised using a scalpel, and no bleeding was noted. Approximately four tumor fragments, each measuring 5–10 mm, were obtained during the biopsy. Intraoperative frozen section analysis was not performed because the macroscopic appearance of the samples indicated sufficient volume and viability. At our institution, frozen section analysis is used selectively when sample adequacy is uncertain; in this case, it was deemed unnecessary. The surgery lasted 54 min, and the amount of bleeding was 1 mL.

**Fig. 5:**
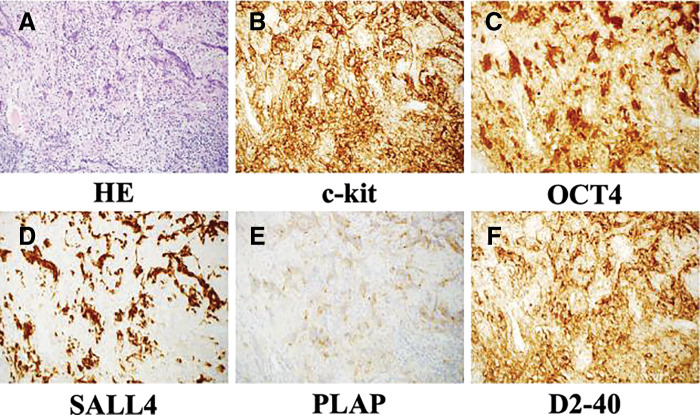
Gross and histopathological findings. (**A**) Hematoxylin-eosin (HE) staining revealed a two-cell pattern with lymphocytic infiltration and epithelioid cell granulomas in the stroma and fibrous tissue (×200). (**B**) Immunohistochemistry was positive for c-KIT (×200). c-KIT positivity highlights the cell membranes of tumor cells, supporting the diagnosis of seminoma. (**C**) Immunohistochemistry was positive for OCT4 (×200). OCT4 positivity is consistent with germ cell origin and helps differentiate from thymoma or lymphoma. (**D**) Immunohistochemistry was positive for SALL4 (×200). SALL4 positivity is highly specific for seminomas and other germ cell tumors. (**E**) Immunohistochemistry was positive for PLAP (×200). PLAP positivity confirms germ cell differentiation, aiding in the distinction from thymic or lymphoid tumors. (**F**) Immunohistochemistry was positive for D2-40 (×200). Negative staining for cytokeratin helps rule out thymic epithelial tumors. HE, hematoxylin-eosin; OCT4, octamer-binding transcription factor 4; PLAP, placental alkaline phosphatase; SALL4, spalt-like transcription factor 4

**Fig. 6:**
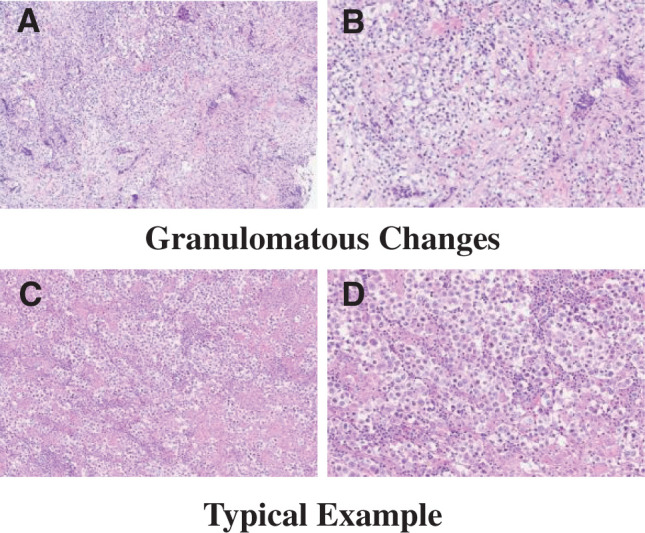
Histopathological findings of granulomatous changes and typical seminoma. (**A**, **B**) Hematoxylin-eosin (HE) staining revealed granulomatous changes in 50%–60% of the entire tumor. Lymphocyte infiltration was comparable to that seen in typical seminoma, but fibrosis was prominent (**A**: ×100, **B**: ×200). (**C**, **D**) HE staining revealed typical findings of seminoma (**C**: ×100, **D**: ×200).

Histopathological examination revealed cells with clear vesicular bodies, lymphocytic infiltration and epithelioid cell granulomas in the stroma, and fibrous tissues with a two-cell pattern. Immunohistochemistry revealed that the tumor was positive for c-KIT, octamer-binding transcription factor 4 (OCT4), spalt-like transcription factor 4 (SALL4), placental alkaline phosphatase (PLAP), and D2-40 (**Fig. 5**). Granulomatous changes were difficult to measure accurately, but were observed in 50%–60% of the tumor. Regarding inflammatory cell infiltration and fibrosis, lymphocyte infiltration was comparable to that of typical seminoma, but fibrosis was more pronounced (**Fig. 6**). Based on histological findings, immunohistochemistry, tumor location, and patient age, a diagnosis of prepubertal seminoma with granulomatous changes was made.

**Fig. 7:**
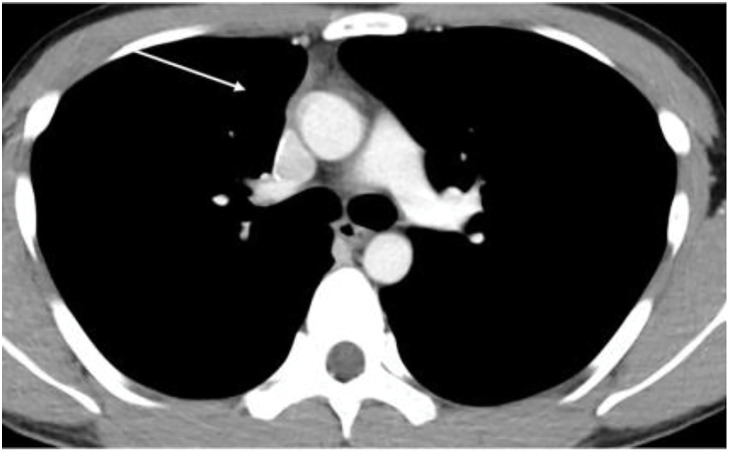
Chest computed tomography findings showing complete tumor disappearance.

No abnormalities were observed in the bilateral testes on ultrasonography. Therefore, the patient was diagnosed with an anterior mediastinal seminoma. The tumor was classified as having a good prognosis, and induction chemotherapy with etoposide, ifosfamide, and cisplatin (VIP chemotherapy) was administered for three courses. Sperm preservation was performed prior to chemotherapy in accordance with our institutional protocol. Complete remission was achieved (**Fig. 7**). The patient has since been monitored every 3 months, with no recurrence or metastasis observed for approximately 2 years.

## DISCUSSION

Most germ cell tumors occur in the testes of young men, while extragonadal primary tumors, such as those in the mediastinum, are relatively rare. Primary mediastinal seminoma is one of the most common types of germ cell tumors. It is generally thought to occur in the anterior mediastinum and is more common in young males.^1,2)^ When a malignant germ cell tumor is suspected in the anterior mediastinum, a testicular origin must be ruled out. Tumor markers, such as α-fetoprotein and β-human chorionic gonadotropin, must be measured before initiating treatment, and a needle biopsy of the tumor must be performed under detailed imaging guidance.^4)^ However, pathological examination is difficult, and misdiagnoses are often reported.^5)^ Similar to testicular seminomas, mediastinal seminomas are malignant tumors that can be cured with multidisciplinary treatment centered on chemotherapy and surgical resection; therefore, a rapid and accurate diagnosis is necessary. Among the various histological challenges, granulomatous inflammation may further complicate diagnosis. Although rare, such reactions have been reported in seminomas arising in both gonadal and extragonadal sites.^1,2)^ These reactions can pose significant diagnostic challenges, especially when extensive, as they may mimic infectious or inflammatory conditions. When granulomatous inflammation dominates the biopsy specimen, there is a risk of misdiagnosing the lesion as sarcoidosis, tuberculosis, or other granulomatous diseases.^3)^ Therefore, clinicians and pathologists should be aware that granulomatous change may occur in both gonadal and extragonadal seminomas, and should consider seminoma in the differential diagnosis when appropriate clinical and radiologic features are present. This case contributes to the understanding of such diagnostic pitfalls and highlights the need for integrating clinical, radiologic, and immunohistochemical findings for accurate diagnosis.

The treatment is generally the same as that for testicular seminoma, with chemotherapy based on the prognostic classification of the International Germ Cell Consensus Classification. For patients in the good-risk category, three courses of bleomycin, etoposide, and cisplatin (BEP chemotherapy) or four courses of etoposide and cisplatin (EP chemotherapy) are recommended. For those in the intermediate- or poor-risk category, four courses of BEP chemotherapy or etoposide, ifosfamide, and cisplatin (VIP chemotherapy) are recommended.^6)^ In this case, the patient was categorized in the good prognosis group with no distant metastasis. However, VIP chemotherapy was chosen because of the possibility of lung surgery following induction chemotherapy,^7)^ and this decision was considered appropriate. Additionally, regarding the management of residual tumors after chemotherapy, as with testicular cancer, resection is recommended if the size of the residual tumor is 3 cm or more.^6,7)^ In this case, because the treatment resulted in complete remission, no additional surgical resection was performed, and careful follow-up was ongoing.

In this case, immunohistochemistry was useful for the accurate diagnosis of mediastinal seminoma. As granulomatous changes were present, scleromyxedema and Hodgkin’s lymphoma were initially considered; however, these were ruled out, and a diagnosis of mediastinal seminoma was made using additional immunohistochemistry. c-KIT is highly expressed in seminoma cells; however, it can also be present in other tumors.^8)^ OCT4 is a specific marker for germ cell tumors and is often negative in non-seminoma cells.^9)^ SALL4 is also positive in seminoma cells and helps differentiate them from undifferentiated germ cell tumors.^10)^ PLAP has high sensitivity for germ cell tumors, including seminomas.^11)^ The results were positive in both cases. Seminomas characteristically express PLAP, SALL4, OCT4, and c-KIT, which are typically negative in thymoma and lymphoma (**Table 1**). SALL4 is especially useful, as it is highly sensitive and specific for germ cell tumors, whereas thymomas and lymphomas do not express this marker.^8–11)^ There have been previous reports where biopsy results raised the suspicion of tuberculosis, leading to the administration of anti-tuberculosis drugs. However, no improvement was observed, and the correct diagnosis was only made after the tumor was removed.^5)^ In the biopsy, the small sample size may have also contributed to the failure in making an accurate diagnosis. As no previous reports of mediastinal seminomas with granulomatous changes exist, the present report may help in the diagnosis of mediastinal seminomas in the future. This case was difficult to diagnose because of the presence of granulomatous changes. However, immunohistochemistry allowed us to reach an accurate diagnosis and choose the most appropriate treatment.

**Table 1:** Immunohistochemical profile of anterior mediastinal tumors: comparison of seminoma, thymoma, and lymphoma

Marker	Seminoma	Thyoma	Lymphoma	Diagnostic utility
PLAP	+	−	−	Specific for germ cell tumors
SALL4	+	−	−	Highly sensitive and specific for seminoma
OCT4	+	−	−	Useful for seminoma; negative in others
c-KIT	+	±	±	Also expressed in thymic carcinoma and lymphoid cells

“+” indicates positive staining; “−” indicates negative staining; “±” indicates partial or weak positivity. The listed markers help distinguish seminoma from histological mimics in small biopsy specimens.

PLAP, placental alkaline phosphatase; SALL4, spalt-like transcription factor 4; OCT4, octamer-binding transcription factor 4

In this case, a biopsy under general anesthesia was useful for a safe and appropriate diagnosis. Mediastinal tumors can cause acute respiratory failure by compressing the trachea or bronchi. In such cases, the use of sedatives or muscle relaxants during tracheal intubation can cause fatal complete tracheal obstruction.^12)^ Additionally, if the bronchial diameter accounts for over one-third of the lumen on CT, the risk of airway obstruction during general anesthesia is extremely high.^13)^ While needle biopsy under local anesthesia is relatively safe, the correct diagnostic rate is only approximately 80%.^14)^ In this case, tracheal compression was not observed, and general anesthesia was administered because the patient was judged to be at a low risk. Additionally, an accurate diagnosis was achieved early by obtaining a sufficient number of tissue samples under thoracoscopic assistance, allowing for the timely selection of appropriate treatment. Although thoracoscopic biopsy is widely used for the diagnosis of anterior mediastinal tumors due to its minimally invasive nature, it may carry a small risk of pleural dissemination, especially in germ cell tumors. By contrast, the Chamberlain procedure, a parasternal mediastinotomy, provides direct access to the anterior mediastinum without violating the pleural space, and is thus considered a safer alternative when pleural seeding must be avoided. In our case, thoracoscopic biopsy was selected based on tumor accessibility and institutional experience, and no pleural dissemination was observed intraoperatively.^15)^ However, the Chamberlain approach remains a valid option, particularly in patients with extensive pleural adhesions or contraindications to thoracoscopy. Based on this case, biopsy under general anesthesia can be considered a safe and useful method for achieving an accurate diagnosis.

## CONCLUSIONS

This case of mediastinal seminoma with granulomatous changes is valuable, as there have been few reported cases of this condition. Immunohistochemistry was useful for the accurate diagnosis of mediastinal seminomas in this case.

## DECLARATIONS

### Funding

This study received no funding from the public, private, or commercial sectors.

### Authors’ contributions

Conceptualization, R. Y.

Writing—original draft preparation, R. Y.

Writing—review and editing, N. U., S. Y., and Y. K.; validation, M. K.

Visualization, M. K. and supervision, M. K.

All authors have read and approved the final version of the manuscript.

### Availability of data and materials

Data sharing is not applicable because no datasets were generated or analyzed in the current study.

### Ethics approval and consent to participate

The study was conducted in accordance with the guidelines of the Declaration of Helsinki and was approved by the Institutional Review Board of Asahikawa Medical University Hospital (No. 24156). Approval date: January 31, 2025. Written informed consent was obtained from the patient to publish this paper.

### Consent for publication

Written informed consent was obtained from the patient for the publication of this study.

### Competing interests

All authors have no competing interests, financial or non-financial.
